# Lin41/Trim71 is essential for mouse development and specifically expressed in postnatal ependymal cells of the brain

**DOI:** 10.3389/fcell.2015.00020

**Published:** 2015-04-02

**Authors:** Elisa Cuevas, Agnieszka Rybak-Wolf, Anna M. Rohde, Duong T. T. Nguyen, F. Gregory Wulczyn

**Affiliations:** ^1^Laboratory F.G. Wulczyn, Institute for Cell and Neurobiology, Charité Universitätsmedizin BerlinBerlin, Germany; ^2^Laboratory S. Sahara, MRC Centre for Developmental Neurobiology, King's College LondonLondon, UK; ^3^Laboratory N. Rajewsky, Max-Delbrück-Centrum für Molekulare MedizinBerlin, Germany

**Keywords:** Lin41, Trim71, ependyma, gene trap, neurogenesis, neural tube closure

## Abstract

*Lin41/Trim71* is a heterochronic gene encoding a member of the Trim-NHL protein family, and is the original, genetically defined target of the microRNA let-7 in *C. elegans*. Both the LIN41 protein and multiple regulatory microRNA binding sites in the 3′ UTR of the mRNA are highly conserved from nematodes to humans. Functional studies have described essential roles for mouse LIN41 in embryonic stem cells, cellular reprogramming and the timing of embryonic neurogenesis. We have used a new gene trap mouse line deficient in *Lin41* to characterize *Lin41* expression during embryonic development and in the postnatal central nervous system (CNS). In the embryo, *Lin41* is required for embryonic viability and neural tube closure. Nevertheless, neurosphere assays suggest that *Lin41* is not required for adult neurogenesis. Instead, we show that *Lin41* promoter activity and protein expression in the postnatal CNS is restricted to ependymal cells lining the walls of the four ventricles. We use ependymal cell culture to confirm reestablishment of *Lin41* expression during differentiation of ependymal progenitors to post-mitotic cells possessing motile cilia. Our results reveal that terminally differentiated ependymal cells express *Lin41*, a gene to date associated with self-renewing stem cells.

## Introduction

LIN41, also known as TRIM71, is a member of the Trim-NHL protein family, and was first described in the nematode *Caenorhabditis elegans* (*C. elegans*) as target of the differentiation-associated microRNA (miRNA) let-7 and therefore part of a heterochronic gene network that controls larval development (Slack et al., 2000; Pasquinelli, 2012; Ecsedi and Grosshans, 2013). The *Lin41* gene is conserved throughout bilateral animals in terms of both amino acid (a.a.) sequence and the presence of binding sites for the miRNAs let-7 and lin-4/miR-125 in the 3′ UTR of the messenger RNA (mRNA). Consistent with the high degree of evolutionary conservation, *Lin41* is essential for the development of many organisms, including fly, frog, zebrafish, and mouse (Slack et al., 2000; Vella, 2004; Kanamoto et al., 2006; Lin et al., 2007; Löer et al., 2008).

As in *C. elegans*, in mouse *Lin41* expression decreases throughout embryogenesis: mouse embryonic stem (mES) cells are *Lin41* positive (Rybak, 2009), and several *Lin41* gene trap mouse lines have been used to report promoter expression in neuroepithelium, facial prominence, branchial arches and limb buds of embryos at developmental day 9.5–10.5 (E9.5–E10.5). Between E10.5 and E12.5, expression gradually declines and no activity has been reported after embryonic stage E13.5 (Schulman et al., 2005; Maller Schulman et al., 2008). Homozygous mutant embryos lacking functional LIN41 present a highly penetrant closure defect of the cranial neural tube. This is detectable from E9.5 on, and does not affect the spinal cord or the most anterior portions of the tube. In addition to the closure defect, knockout embryos cease development and die between E9.5 and E11.5, although the cause of embryonic lethality has not yet been defined (Maller Schulman et al., 2008; Chen et al., 2012). Embryonic lethality has precluded the study of LIN41 function at later stages; nevertheless, after birth *Lin41* expression has been reported in the germinal layer of the spermatogonial stem cells of mouse testis, in the interfollicular stem cells of the epidermis and in ciliated epithelium of the male and female reproductive tract. As in the embryo, LIN41 expression displayed a reciprocal relationship to the let-7 miRNA in these adult stem cell niches (Rybak et al., 2009), and has therefore been considered a gene associated with proliferation and undifferentiated cell types. To date, neither the presence nor the potential function of *Lin41* in the postnatal central nervous system (CNS) has been investigated.

Recent studies have begun to address the molecular functions of LIN41. Like other members of the Trim-NHL family, the LIN41 protein was demonstrated to have RING-dependent ubiquitin ligase activity (Rybak et al., 2009), reviewed in Wulczyn et al. (2011). LIN41 was found to localize to cytoplasmic P-bodies and directly interact with the miRNA pathway proteins Argonaute 2 (AGO2) and DICER, and to repress miRNA activity by promoting degradative ubiquitination of AGO2 (Rybak et al., 2009; Chen et al., 2013). In particular, LIN41 was found to cooperate with the pluripotency factor LIN28 to suppress activity of the pro-differentiation miRNA let-7 (Rybak et al., 2009). In *C. elegans*, similar regulatory interactions between LIN41, the Argonaute protein ALG-1 and let-7 have been shown to mediate changes in the regenerative capacity of neuronal axons that take place during development of the nervous system (Zou et al., 2013). Nevertheless, the physiological relevance of LIN41-mediated AGO2 ubiquitination has been challenged (Chang et al., 2012; Chen and Wichterle, 2012; Loedige et al., 2012). In a study of LIN41 function in the neuroepithelium of LIN41 mutant mice, Chen et al., identified SHCBP1, a novel component of the fibroblast growth factor (FGF) signaling pathway, as an interaction partner of LIN41 and target for non-degradative polyubiquitination. Stabilization of SHCBP1 by LIN41 was associated with an increased proliferative response to FGF signals in the developing embryo (Chen et al., 2012). In another line of investigation, the NHL domain of LIN41 was shown to mediate RNA-binding in mES cells (Kwon et al., 2013). This is likely related to the involvement of LIN41 in the post-transcriptional regulation of the CDKN1A (Chang et al., 2012), RBL1 and RBL2 (Loedige et al., 2012) or EGR1 mRNAs in ES cells (Worringer et al., 2013). In each of these studies candidate substrate mRNAs were identified based on a combination of physical association and reduced abundance in the presence of LIN41.

The adult mammal brain retains two neurogenic niches, the dentate gyrus of the hippocampus and the subventricular zone (SVZ) of the lateral ventricles (Reynolds and Weiss, 1992; Richards et al., 1992; Gage et al., 1995). SVZ stem cells are interposed between the ependymal cells that line the ventricular surface, and the role of this cellular arrangement in the maintenance of the niche has just begun to be elucidated (Paez-Gonzalez et al., 2011). Ependymal cells are born directly from radial glia, following a caudo-rostral pattern, mainly during embryonic stages after E14.5. However, they remain undifferentiated until the second week after birth, when they start acquiring their particular characteristics such as motile multicilia (Spassky et al., 2005). Recently, an en-face technique has been used to describe the three-dimensional architecture of the neurogenic adult stem cell niche (Mirzadeh et al., 2010). Within the niche ependymal cells are distributed in the ventricle wall in a so-called pinwheel pattern surrounding a single GFAP-positive neural stem cell, (also known as type B cell). This arrangement allows each stem cell to retain direct contact to the ventricle through its apical primary cilium and suggests a structural/supporting role for ependymal cells within the niche. Moreover, the assembly and integrity of the ependymal pinwheel is essential to maintain the ability of type B cells to proliferate and generate new neurons (Mirzadeh et al., 2008). Ependymal cells are known to act as neural stem cells in chordates (Horie et al., 2011), and latent stemness has been reported for the cells lining the central canal of the rodent spinal cord (Sabelström et al., 2014). Although still debated (Coskun et al., 2008; Gleason et al., 2008; Carlén et al., 2009), the majority of studies in the mammalian telencephalon indicate that the ependyma is post-mitotic and does not normally make a direct contribution to neurogenesis (Spassky et al., 2005; Gómez-Roldán et al., 2008; Luo et al., 2008; Mirzadeh et al., 2008; Danilov et al., 2009; Giachino and Taylor, 2009). Nevertheless, ependymal cells are likely to play at least indirect roles in adult cortical neurogenesis both as conduits of trophic support and as important structural elements of niche architecture (Lim et al., 2000; Kuo et al., 2006; Sawamoto et al., 2006; Gajera et al., 2010; Lavado and Oliver, 2011; Paez-Gonzalez et al., 2011), reviewed in Jiménez et al. (2014).

Given the known LIN41 expression in embryonic stem cells and the defect in embryonic neurogenesis in null mice, we were interested in possible functions for LIN41 in adult stem cell niches in the brain. For this purpose, we have generated an independent gene trap mouse line for *Lin41*, to study regulation of the *Lin41* promoter in adult tissues and to serve as a source for genetically tagged cells for *in vitro* culture. The *Lin41* expression pattern and deletion phenotype of our line is similar to previous reports, with no embryos surviving past E12.5 and a completely penetrant defect in neural tube closure. Using this model, we show that after a period of absence in late stages of embryogenesis and early postnatal development, *Lin41* expression resumes in the ventricular zones of the mouse brain at the level of promoter activity and protein expression. Whereas neurospheres derived from the subventricular zone lack *Lin41* promoter activity and LIN41 protein, the timing and localization of LIN41 expression matches the period of ependymal cell maturation and marker acquisition. Performing immunostaining of coronal slides and whole mount ventricular preparations with several ependymal markers, we confirm the ependymal identity of LIN41-positive cells in the adult neurogenic niche. Using primary ependymal cell culture, we demonstrate that multiciliated ependymal cells acquire LIN41 during differentiation. LIN41 has been shown to promote stem cell plasticity in a number of developmental contexts (Worringer et al., 2013; Spike et al., 2014; Tocchini et al., 2014). The inverse relationship between *Lin41* expression and terminal differentiation observed in the adult neurogenic niche raises the question of possible new functions for this ancient regulator.

## Materials and methods

### Gene trap mouse

All experiments were conducted according to the European and German laws, following the Animal Welfare Act and the European legislative Directive 86/609/EEC, followed by Directive 2010/63/EU from 2010 on and updated in 2013 and the regulations of the Animal Welfare Committee of the Charité, Berlin. The number of sacrificed animals and their stress or discomfort was kept to a minimum. The animal welfare committee of Charité, Berlin, approved and supervised the experiments performed in the present work. The experimental license number for the procedures used is T01012/11.

Embryonic stem cells for the *Lin41* gene trap mouse line *Lin41^Gt(lacZ−neo)fgw^*, henceforth referred to as *Lin41^gt^*, were obtained from the German Gene Trap Consortium. TBV2 mES cells (derived from 129S2 strain) carrying an insertion of the FlipROSAbgeo0 vector within the second intron of *Lin41* were injected into C57/Bl6/J blastocysts in collaboration with Dr. Boris Jerchow at the Max Delbrück Center for Molecular Medicine in Buch, Germany. Chimeras were genotyped using the following primers: Intron2 Fw: (TTC TGC ATC CAG AGT GCA AC) and Intron2 Rev: (CTT CCT CTG ACC TTT GCT GG) or Gene Trap Rev: (ACC AGC TGT GCG CAT AGT G). See: http://www.informatics.jax.org/javawi2/servlet/WIFetch?page=alleleDetail&key=91390#imsr for the gene trap insertion site based on Splinkerette PCR and 5′ RACE. Mice from the gene trap strain were backcrossed against C57/Bl6/J for at least five generations to ensure homogeneity of genetic background before conducting experiments.

For embryonic study, heterozygote *Lin41^+/gt^* mice were mated and a positive vaginal plug was considered as day post-conception, or embryonic day, 0.5 (E0.5). At the designated embryonic day, pregnant females were sacrificed by cervical dislocation, decidua removed and washed in ice cold PBS. Each embryo was dissected from the decidua with the yolk sac used for genotyping by extracting DNA in 200 μ l lysis buffer (25 mM NaOH, 0.2 mM EDTA) for 40 min at 80°C, followed by neutralization with 200 μ l 40 mM Tris buffer pH 5.0.

### LacZ detection

Whole embryos were fixed in fixation buffer (5 mM EGTA, 0.2% Glutaraldehyde in PB 0.1 M 70 mM Na_2_HPO_4_, 30 mM NaH_2_PO_4_) O/N at 4°C and then washed three times for 15 min in washing buffer (PB, 2 mM MgCl_2_). They were exposed to staining buffer (PB, 2 mM MgCl_2_, 5 mM K_4_Fe(CN)_6_ (Sigma,P9287), 5 mM K_3_Fe(CN)_6_ (Sigma,P8131) and 1 mg/ml X-Gal UltraPure (Invitrogen, B-1690) O/N at 37°C, and the reaction was stopped by removing buffer and washing three times with PB buffer.

### Western blot

Embryos were snap frozen in liquid nitrogen and disrupted with a mortar and pestle; the resulting powder was resuspended in TNN lysis buffer (50 mM Tris pH 7.4, 150 mM NaCl, 0.5% NP-40 (Igepal), 5 mM EDTA, 1x Protease Inhibitor Cocktail Set I (Calbiochem, 539131-10VL) using approximately 1 ml buffer/mg of tissue. Tissue or pelleted mouse embryonal carcinoma (P19) cells were incubated 20 min on ice after TNN resuspension. Lysates were cleared by centrifugation for 20 min at 4°C and 14,000 rpm and supernatants transferred to a fresh tube. Protein concentration was measured using the Bio-Rad Protein Assay Dye Reagent (Bio-Rad, 500-0006) and a standard curve of BSA (0, 0.25, 0.50, 1 μg/ml). 25 μ g protein extract were subjected to 8% SDS-PAGE and transferred to Immobilon-P Transfer Membrane membranes (Millipore, IPVH00010). Antibodies: polyclonal anti-LIN41 serum antibody 1:4000 (made in rabbit, Pineda Antibodies Service, Berlin) (Rybak et al., 2009), mouse monoclonal GFAP (Sigma, G3893 1:1000) and mouse monoclonal Vinculin (Sigma, V9231) as loading control.

### Immunostaining

Young adult mice (6–8 weeks old) were administered a lethal dose of Avertin solution (5 mg/ml of 2, 2, 2-Tribromoethanol in tert-Amyl Alcohol) via intra-peritoneal injection. Animals were subjected to intracardiac perfusion with PB, followed by 30 ml 4% PFA to fix the brain tissue. After perfusion, brains were post-fixed in 4% PFA solution O/N at 4°C, and then submerged in a 30% sucrose solution in 1x PBS O/N at 4°C for cryoprotection followed by embedding in O.C.T. compound (Leica, 3808610E) and freezing on dry ice. Tissue blocks were stored at −80°C and transferred to a Leica Cryostat at −20°C to section 12 μm slices.

Tissue was post-fixed with 4% PFA for 10 min at R/T and then washed three times for 5 min with 1x PBS. A permeabilization and blocking step followed in blocking buffer (1x PBS, 5% normal goat serum and 0.2% Triton-X) for 1 h at R/T. Sections were incubated in a moist, Parafilm covered chamber with primary antibodies diluted in blocking buffer O/N at 4°C. The following day they were washed three times with 1x PBS and then incubated with secondary antibodies in blocking buffer for 1 h at R/T. After washing three times in 1x PBS the sections were mounted on a slide with DAPI or DRAQ5 to counterstain the nuclei in fluoro-protective mounting medium, covered with a glass coverslip and let dry at R/T horizontally, protected from the light.

To avoid cross-reaction between the secondary antibodies in co-staining of CD133 (made in rat) with Lin41 (made in rabbit), the protocol was performed sequentially: an incubation of rabbit anti-Lin41 and its secondary antibody goat anti-rabbit, followed by an incubation of rat anti-CD133 and its secondary rabbit anti-rat. Images were acquired with an epifluorescent Olympus BX51 microscope using Magnafire software or a Leica SL confocal microscope and processed using Adobe Photoshop CS4 for Mac.

Primary antibodies used were: anti-mLin41 serum, rabbit, peptide, targeting the NHL sequence, (Pineda Antibodies Service, Berlin) (Rybak, 2009)1:50, mouse monoclonal GFAP (Sigma, G3893 1:400), mouse monoclonal acetylated Tubulin (Sigma, T6793, 1:500), rat monoclonal CD24 (Abcam, ab64064 1:250), rat monoclonal CD133 (eBioscience, 14-1331 1:500), mouse monoclonal FoxJ1 (eBioscience, 14-9965 1:750), and then treated with secondary antibodies from Molecular Probes (1:1000).

### Brain lateral ventricle wall whole mount preparation

The protocol was performed as described in Mirzadeh et al. (2010). Briefly, adult mice were sacrificed by cervical dislocation and brains were isolated and sectioned in two halves to separate the hemispheres. A coronal cut was made posteriorly to expose the hippocampus, which was then pulled away to reveal the lateral wall overlying the striatum. The cortex and corpus callosum were dissected off, resulting in a section of uniform thickness. The tissue was fixed and treated using the standard protocol for immuno- or X-Gal staining. Jellifying and fluoro-protective mounting medium DABCO (Sigma, 10,981) was used due to the thickness of the preparation, and the sample was allowed to settle at least O/N before imaging.

### Neural stem cells derived from adult SVZ

The culture of neural stem cells was performed as previously described (Reynolds and Weiss, 1992; Reynolds et al., 1992). Briefly, mice from 6 to 8 weeks old were sacrificed by cervical dislocation and the brains were removed in ice-cold 1x PBS. Two or three coronal sections containing the medial zone of the lateral ventricles were made with a scalpel, and the SVZs were dissected with 20 G needles. The tissue was mechanically dissociated with the needles and digested with an enzymatic solution containing 0.9 mg/ml papain (Worthington, 3119), 0.2 mg/ml L-Cystein (Sigma, C-8277), and 0.2 mg/ml EDTA (Sigma, E-6511) in HBSS medium (Gibco, 24020-091) for 30 min at 37°C.

The tissue was centrifuged 5 min at 1000 rpm, the enzymatic solution was aspirated and cells mechanically dissociated and plated in 10 cm dishes, one brain per plate, in Complete Adult NeuroCult™ Proliferation Medium (450 ml NeuroCult® NSC Basal Medium, StemCell Technologies 05700 plus 50 ml NeuroCult® NSC Prolif. Supplements, StemCell Technologies 05701) supplemented with 20 ng/ml EFG (Peprotech, AF-100-15), 20 ng/ml bFGF (Peprotech, 100-18B) and 0.7 U/ml heparin (Sigma, H3149). After 4–5 days neurosphere formation was observed. Following a three min 0.05% Trypsin-EDTA (Gibco, 25300-054) digestion and mechanical dissociation cells were passaged at 50000 cells/ml and experiments were performed at passages two to five.

### Ependymal cell culture from newborn forebrain

Ependymal primary culture was performed as previously described (Guirao et al., 2010; Paez-Gonzalez et al., 2011). Prior to dissection, 25 cm^2^ flasks were coated with 40 μg/ml Poly-L-Lysine (Sigma, P1524) 1 h at 37°C, then washed three times with MilliQ water and dried 1 h in sterile conditions. Newborn mice (P0 to P2) were sacrificed by decapitation and brains were isolated in ice-cold 1x PBS and dissected, discarding meninges, olfactory bulb and hippocampal formation. The remaining tissue was mechanically dissociated using a P-1000 pipette, and digested in 1 ml ependymal enzyme solution (DMEM containing 10% FBS; 0.9 mg/ml Papain, Worthington, 3119; 1% DNaseI Worthington, 2139 and 12 mg/ml L-Cystein, Sigma, C-7352) per brain for 45 min at 37°C. The reaction was stopped with 1 ml stop solution (Liebovitz's L-15 medium, Gibco 11415, containing 10% FBS, 1% DNaseI Worthington, 2139 and 12 mg/ml L-Cystein, Sigma, C-7352) followed by three rounds of centrifugation at 1000 rpm and mechanical dissociation in Leibovitz's L 15 medium. Cells were plated in Poly-L-Lysine coated 25 cm^2^ flasks (one brain per flask) and incubated 3–5 days at 37°C, 5% CO_2_, 95% humidity until the culture reached confluence. When confluent, flasks were shaken O/N at 250 rpm at R/T. The next day cells were de-attached using Trypsin and 20 μ l drops containing 1.5 × 10^5^ − 2 × 10^5^ cells were plated on 15 mm glass coverslips in 24 well plates to yield a confluent culture. After 1 h incubation at 37°C, 5% CO_2_, 95% humidity, 1 ml medium (DMEM, 10% FBS, 1% P/S) was added. Next day, medium was changed to differentiation medium DMEM, 1% P/S, and cells were incubated for 10–15 days (Paez-Gonzalez et al., 2011).

### RNA isolation

RNA was isolated from P19 cells or tissue using TRIzol reagent (Ambion, 15596026) according to manufacturer's instructions and stored at −80°C.

### cDNA synthesis

2 μl dNTP 10 mM, 4 μl 5x RT buffer, 0.5 μl Thermo Scientific Ribolock RNAse inhibitor, 1 μl RevertAid™ Premium Reverse Transcriptase (Fermentas, EP0733) were added to a solution of 5 μ g RNA and 1 μl OligodT 100 μM in 12.5 μ l. The reaction was incubated for 30 min at 50°C followed by inactivation at 85°C for 5 min.

### PCR genotyping

PCR was performed using a PXE 0.2 Thermal Cycler (Thermo Scientific), in 0.2 ml tubes. Lin41 E2-RT Fw (CTG TGA CAC CTG CTC TGT CC), Lin41 E2-RT Rev (GAA AGA CCG CGA AGA GTT TG), ß-Actin Fw (GGC TGT ATT CCC CTC CAT CG) and ß-Actin Rev (CCA GTT GGT AAC AAT GCC ATG T) primers were annealed at 56°C yielding a diagnostic 420 bp fragment.

## Results

### Embryos deficient in Lin41 display a failure in neural tube closure and lethal phenotype at E9.5

**Figure 1:**
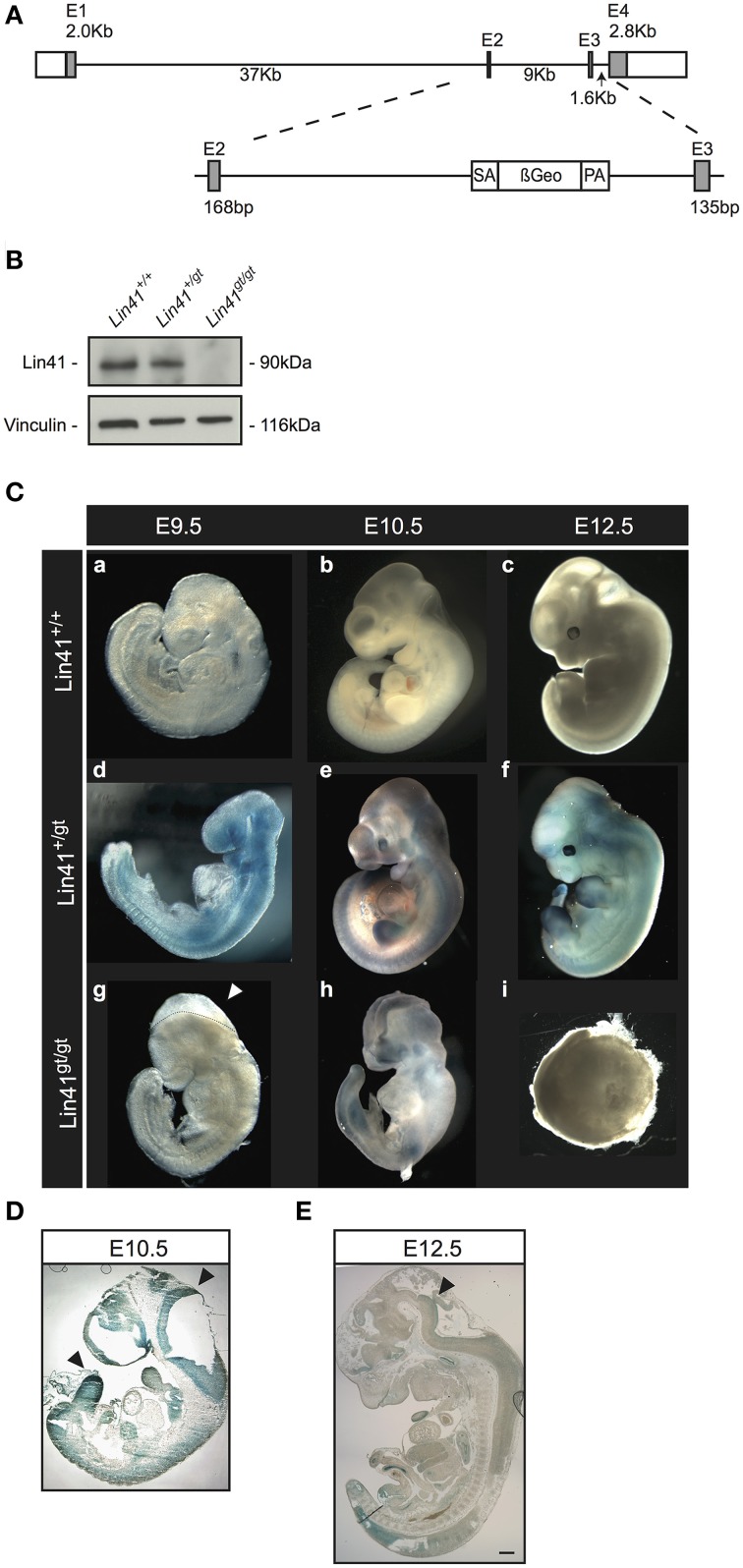
**Characterization of a *Lin41* gene trap mouse line**. **(A)** Scheme of the wild type *Lin41* gene and the gene-trap cassette insertion in the second intron. **(B)** Western blot from whole E9.5 embryo lysates with genotypes indicated above each lane. The filter was developed with anti-Lin41 and anti-Vinculin antibodies as loading control (left). **(C)** X-Gal staining of wild type, heterozygote, and homozygote knockout embryos, from E9.5 to E12.5. Knockout animals display a failure in neural tube closure and die after E9.5 (panel g: *Lin41^gt/gt^* E9.5 embryo not stained, dotted line and arrowhead indicate the open neural fold), encapsulating and reabsorbing by E12.5. Embryos not to scale. **(D)** Sagittal E10.5 *Lin41^+/gt^* mouse embryo section, X-Gal stained; neuroepithelium and limb buds are strongly positive (arrowheads). **(E)** Sagittal E12.5 *Lin41^+/gt^* mouse embryo section; vestiges of X-Gal signal remain in the neuroepithelium (arrowhead).

Our mouse line was generated using a mES cell line obtained from the German Gene Trap Consortium that carries a gene trap vector insertion in the *Lin41* gene. Splinkerette PCR analysis confirmed the insertion site of the gene trap cassette in the second intron at position 7957 (Figure 1A). The translated mRNA is predicted to generate a chimeric protein with LIN41 truncated after a.a. 327, resulting in replacement of part of the Coiled-Coil and the entire Filamin and NHL domains with the β-Geo reporter protein.

We performed western blot analysis on extracts from E9.5 embryos to confirm the loss of LIN41 wild type protein in homozygote gene trap mutants (Figure 1B, compare Lanes 1 and 3). For heterozygotes, the protein levels of LIN41 were reduced to approximately one-half that of the wild type (Figure 1B, compare Lanes 1 and 2).

We examined embryonic stages from E9.5 on and observed that heterozygote animals (*Lin41^+/gt^*) do not present any visible phenotype, size change or other abnormality in comparison to the wild type animals (*Lin41^+/+^*) (Figure 1C, panels a–f). Nevertheless, the homozygote mutant embryos stop developing at around E9.5 and die shortly thereafter (Figure 1C, panels g–i). Additionally, the lethal phenotype is accompanied by a highly penetrant closure defect in the anterior craniofacial region of the neural tube, corresponding to the forebrain/midbrain boundary, while the spinal cord and the rostral extremity of the forebrain are not affected in *Lin41* deficient embryos (Figure 1C, panels g,h, closure defect marked by dotted line and arrowhead in g). When embryos were observed at later stages, up to E13.5, knockout animals were degenerating, often encapsulated and undergoing reabsorption (Figure 1C, panel i).

Taken together, our results confirm and augment the previous evidence that *Lin41* is essential for mouse embryonic development. We then took advantage of the reporter gene β-Gal, to study *Lin41* promoter activity in *Lin41*^+/*gt*^ embryos and the consequences of the loss of only one copy of functional *Lin41* gene, and gain insight into its expression.

### Early to mid-embryonic expression of Lin41

The reporter gene *LacZ* in the gene trap vector was used to track the activity of the *Lin41* promoter via X-Gal staining during embryonic development, starting at E9.5. At this stage, staining is prominent in the entire embryo, with the exception of the primordial cardiac sac (Figure 1C, panel d). Afterwards, the *Lin41* promoter activity decreases rapidly: at E10.5 X-Gal signal can be detected within the neuroepithelium, branchial arches, spinal cord, somites, limb, and tail buds (Figure 1C, panel e and Figure 1D).

In particular, at the onset of CNS development, the neuroepithelium shows a prominent staining between E9.5 and E10.5 (Figure 1C panels d,e, and Figure 1D). Thereafter X-Gal positive cells are unevenly distributed in a progressively thinner layer along the inner surface of the ventricle (Figure 1E). In a comparable fashion, the bumps corresponding to the nascent limb buds around E10.5 are intensely stained. Shortly thereafter, as the limb buds emerge from the body and expand, X-Gal staining declines and is limited to the most distal surface at E12.5. (Figure 1C panels e,f, Figures 1D,E and Supplementary Figure 1A). Subsequently the signal progressively declines; at E12.5 X-Gal signal is present in the same structures, but is spatially more restricted (Figure 1C, panel f). At E13.5 it is no longer possible to identify cells positive for *Lin41* promoter activity in either the developing nervous system, the limb or the tail buds (see Supplementary Figure 1B and data not shown).

These results confirm previous observations and provide additional details regarding the dynamic activity of the *Lin41* promoter and its downregulation in the neuroepithelium between E9.5 and E13.5. The known post-embryonic expression of *Lin41* in a number of stem cell niches including the skin and testes (Rybak et al., 2009) prompted us to examine Lin41 in postnatal CNS development.

### *Lin41* is expressed in the postnatal CNS in the ventricular walls of the brain

**Figure 2:**
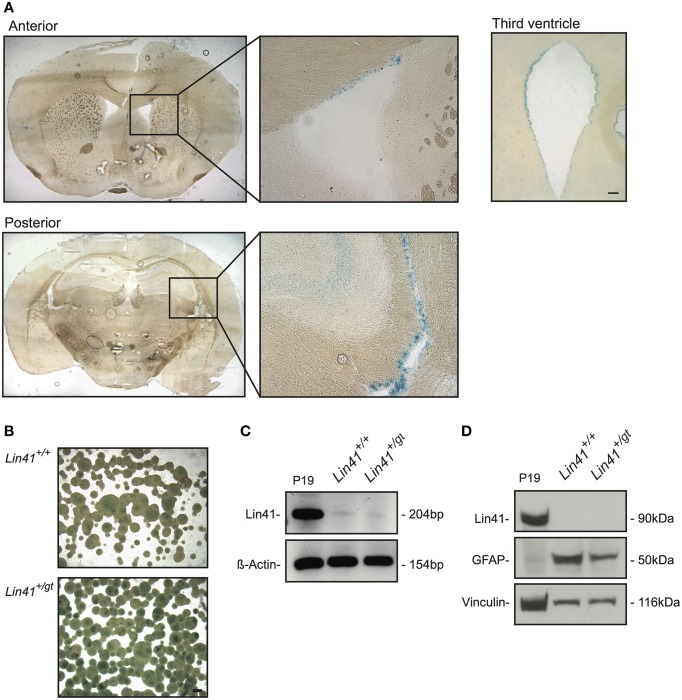
***Lin41* is present in the adult SVZ but not SVZ-derived neurospheres (NSPs)**. **(A)** X-Gal staining of anterior and posterior coronal sections of an adult heterozygote brain, boxes indicate fields containing the lateral ventricle shown at higher magnification (right). Diffuse staining of the hippocampus most likely reflects background and not specific X-Gal staining (see Supplementary Figure 1D). A view of the third ventricle is also provided for comparison (scale bar 100 μm). **(B)** X-Gal staining of NSPs cultured from wild type and heterozygote SVZs. Scale bar 100 μm. **(C)** RT-PCR from cDNA of NSP and positive control P19; ß-Actin was used as loading control. **(D)** Western blot of wild type and heterozygote NSPs and positive control P19 lysates as indicated; antibodies and their corresponding signals are indicated on the left (Vinculin serves as loading control).

To date, expression of *Lin41* in the adult CNS has not been reported. Using heterozygote gene trap animals we detected *LacZ* activity exclusively in cells lining the wall of the four ventricles of the postnatal brain (Figure 2A). Within the lateral ventricles, cells positive for *Lin41* promoter activity were found along the lateral, medial and superior walls. Additionally, the third and the fourth ventricles appeared to be lined by a continuous layer of *Lin41* positive cells (Figure 2A, and data not shown for fourth ventricle). We failed to detect *Lin41* positive cells in any additional structure of the brain other than the ventricles. This pattern of *Lin41* promoter activity was observed as early as postnatal day 10 (P10) and was maintained throughout adulthood, as it is present in animals 9 months and older (Figure 2A, Supplementary Figures 1C,D and data not shown).

### Adult neural stem cells *in vivo* and *in vitro* do not express *Lin41*

To characterize the cell types expressing *Lin41* in the ventricular walls we began by determining their neurogenic potential in an *in vitro* neurosphere assay (Reynolds et al., 1992; Reynolds and Weiss, 1996). In this assay subependymal progenitors but not ependymal cells themselves generate proliferating cell clones that can be cultured as neurospheres. We used 4–6 week old wild type and heterozygote littermates from the *Lin41* gene trap line and dissected tissue from the SVZ to establish a primary stem cell culture. The cells were maintained in culture for two to five passages to ensure purity and homogeneity of the neurospheres.

*Lin41* promoter activity and expression in adult neurospheres was assessed using X-Gal staining, RT-PCR and western blot (Figures 2B–D). The X-Gal reaction is positive in the heterozygote cultures, which present a blue precipitate staining absent in the wild type sample (the apparent signal in the center of large wild type spheres reflects background) (Figure 2B). Despite that positive reaction, the RT-PCR amplification of *Lin41* mRNA yielded only a faint band in samples from either wild type or heterozygote (Figure 2C). Moreover, at the protein level we failed to detect a band with anti-Lin41 antibody at the appropriate size in western blots (Figure 2C). These results suggest that postnatal neural stem cells in the SVZ do not express *Lin41*, despite the characteristic expression of *Lin41* in embryonic neural progenitors. We therefore hypothesized that *Lin41* expression within the SVZ may be restricted to ependymal cells.

### *Lin41* is expressed in ependymal cells of the brain

**Figure 3:**
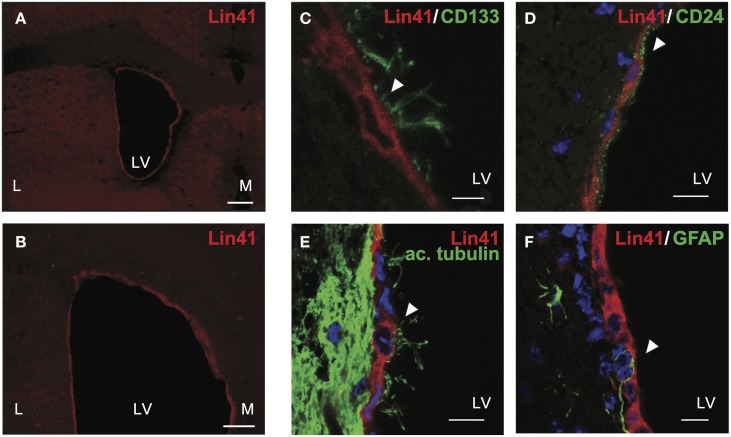
**LIN41 protein is expressed in ependymal cells**. **(A–F)** Immunostaining of adult wild type brain coronal sections with antibodies for Lin41 (red) with overview of lateral ventricles **(A,B)** and higher magnification co-staining with ependymal markers as follows (green) CD133 **(C)**, CD24 **(D)**, acetylated α-tubulin (ac. tubulin, **E**), and GFAP **(F)**. Scale bars 10 μm except **(C)**, 5 μm. Arrowheads indicate double positive cells **(C–E)** or GFAP negative cell **(F)**. L, lateral; LV, lateral ventricle; M, medial.

To address the identity of X-Gal positive cells in the ventricular walls of *Lin41^+/gt^* animals, we correlated *Lin41* promoter activity with protein expression. Immunostaining was performed using an antibody against LIN41 designed to recognize a peptide sequence within the NHL-repeat region. Cytoplasmic LIN41 staining was detected in the apical layer of cells surrounding the lateral ventricles, suggesting a high degree of overlap between promoter activity and protein expression (Figures 3A,B).

To further characterize the identity of the LIN41 positive cells we performed co-staining analysis with lineage markers. Both the apical position of the cells and the developmental timing of *Lin41* promoter activity in the SVZ suggested that ependymal cells might express *Lin41*. To relate LIN41 positive cells to multiciliated ependymal cells we used a panel of classic markers for co-staining: CD133 (also referred to as Prominin-1) is a glycoprotein expressed in different cell types in the adult organism including neuroepithelial progenitors and ependymal cells (Pfenninger et al., 2007), and CD24 is an adhesion molecule present in ependymal cells but not the underlying GFAP-positive astrocytes (Calaora et al., 1996). Additionally, we used acetylated α-tubulin to label the cytoskeletal structure of the cilia and GFAP as an astrocyte marker (Pfenninger et al., 2010). LIN41 positive cells in the lateral ventricles were also positive for CD133 (Figure 3C), CD24 (Figure 3D), acetylated α-tubulin (Figure 3E), but not for GFAP (Figure 3F). Taken together, these data support the hypothesis of the ependymal identity of LIN41-positive cells in the postnatal CNS.

**Figure 4:**
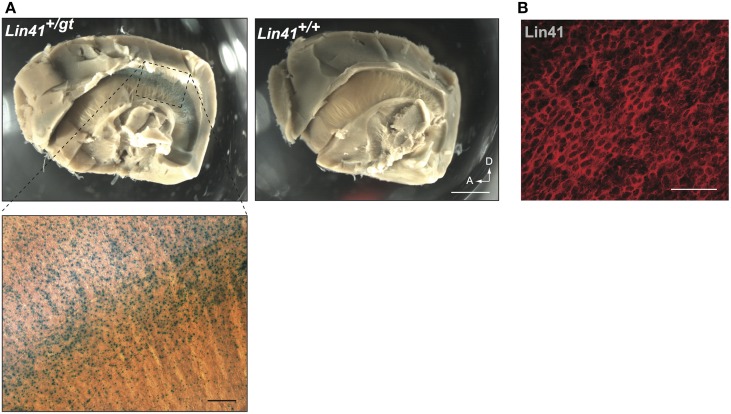
**Whole mount lateral ventricle preparation**. **(A)** Whole mount of adult brain from heterozygote and wild type mice stained for X-Gal (blue). Arrows indicate dorsal and anterior coordinates. Scale bar 1 mm. Bottom left panel shows higher magnification view of the X-Gal staining in the lateral wall of the ventricle (scale bar 100 μm). **(B)** Immunostaining of Lin41 (red) in a wild type sample prepared in parallel. Scale bar 100 μm.

To address in more detail the promoter activity of *Lin41* in the ventricular zone, we took advantage of the recently developed whole mount procedure, which allows visualization of the apical face of the lateral wall of the lateral ventricle (Mirzadeh et al., 2008). Employing X-Gal staining, widespread expression of *Lin41* can be detected on the surface of the lateral ventricle wall of *Lin41^+/gt^* animals (Figure 4A). Immunostaining with anti-Lin41 antibody confirms this expression pattern, and reveals that the protein is uniformly distributed in the cell cytoplasm (Figure 4B).

### *Lin41* is expressed in ependymal cells *in vitro* that display motile multicilia

**Figure 5:**
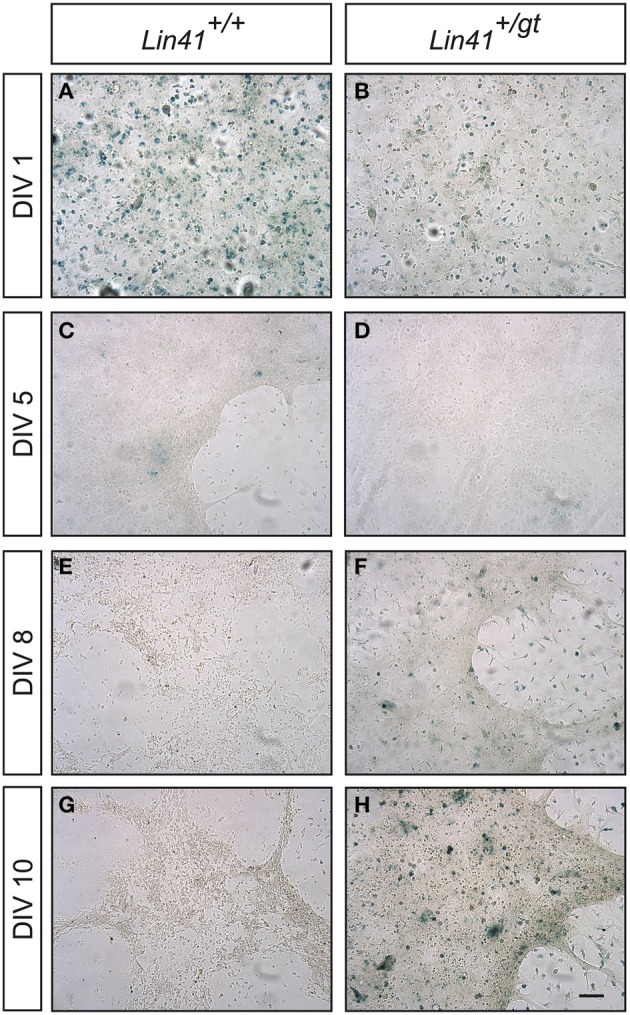
***Lin41* promoter activity in primary ependymal cultures**. X-Gal staining of cultures derived from wild type (left column, panels **A,C,E,G**) and heterozygote (right column, panels **B,D,F,H**) newborn brain tissue are compared. Time in culture is expressed in days *in vitro* (DIV, left). Scale bar 100 μm.

To further study how *Lin41* expression was modulated during ependymal differentiation we took advantage of a primary culture protocol recently developed to allow modeling of ciliogenesis and the cilia orientation program (Guirao et al., 2010; Paez-Gonzalez et al., 2011). Cells isolated from the newborn brain are first expanded during a proliferative phase in culture followed by a differentiation phase in which ciliogenesis occurs, thus resembling the time course of ependyma generation in the brain. We established a primary culture of ependymal cells from gene trap heterozygote (*Lin41^+/gt^*) and wild type (*Lin41^+/+^*) littermates, using X-Gal staining at different time points to monitor promoter activity (wild type being the negative control). *Lin41* transcription is induced upon ependymal differentiation at day *in vitro* 8 (DIV8), as reflected in the heterozygote culture that starts to show X-Gal positive cells (Figures 5E,F compared to Figures 5C,D). The number of positive cells increases with time in culture and comprises the majority of cells after 10 days in culture (Figures 5G,H). As expected, the wild type culture remains negative throughout differentiation. The blue cells observed on the first day in both cultures likely correspond to contaminating cells derived from the choroid plexus that have endogenous ß-Gal activity, and rapidly disappear from the culture under differentiation conditions (Figures 5A,B) (Abeliovich et al., 1992).

**Figure 6:**
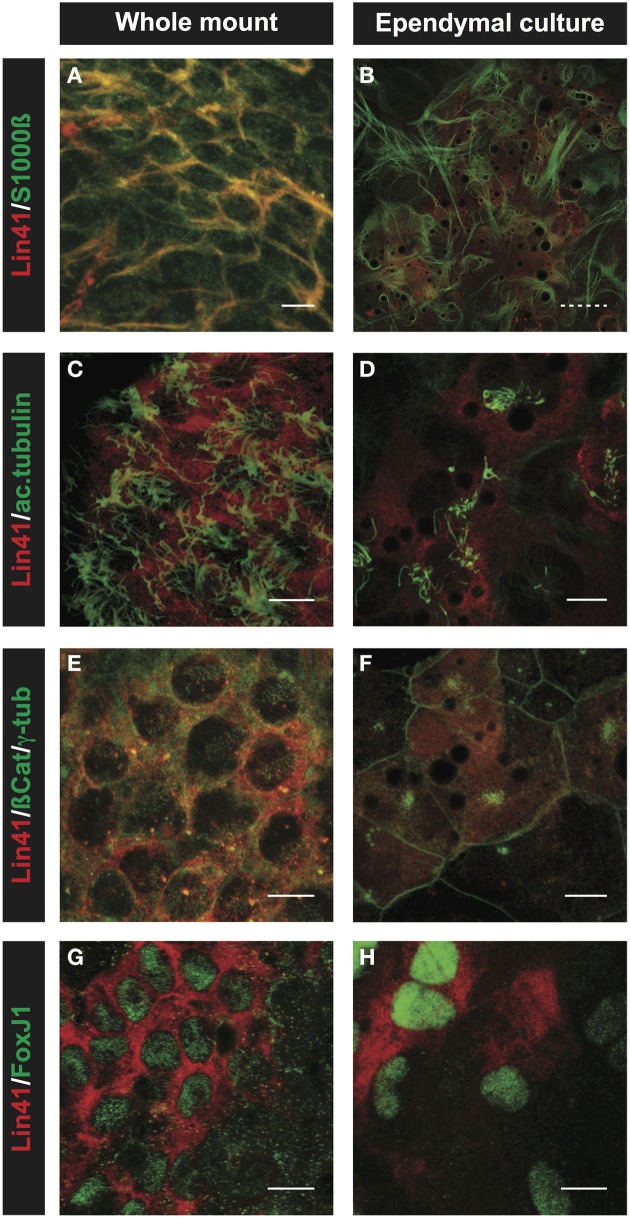
**Comparison of ependymal LIN41 expression in whole mount immunostaining and primary cell culture**. Immunostaining of lateral wall whole mounts from adult wild type mouse brain (left column) and primary ependymal culture derived from newborn brain tissue at DIV15 (right column). LIN41 immunostaining is in red, marker antibodies in green. S100ß is an ependymal marker **(A,B)**, acetylated α-tubulin (ac.tubulin) stains the cilia **(C,D)**, ß-Catenin is used to label the cell-cell contacts and γ-tubulin the basal bodies of the cilia **(E,F)**. FoxJ1 is a nuclear transcription factor responsible for the multicilia differentiation program **(G,H)**. Scale bar 10 μm except for **(B)**, with dashed line 40 μm.

Immunostaining with a panel of specific antibodies was used to confirm the ependymal identity of the primary culture *in vitro* in comparison to the whole mount preparations from the lateral ventricle (Figure 6). For these experiments we chose S100ß and FoxJ1 as ependymal lineage markers (Didier et al., 1986; Jacquet et al., 2009), and used acetylated α-tubulin and γ-tubulin to visualize the cytoplasmic axoneme and basal bodies of cilia, respectively (Muresan et al., 1993). After 5 days in culture, *in vitro* primary cultures display strong S100ß and LIN41 staining (Figures 6A,B), consistent with ependymal identity. Furthermore, central clusters of long cilia projecting from the apical surface of LIN41^+^ cells can be seen in both the whole mount and primary culture preparation after staining for acetylated α-tubulin (Figures 6C,D). This can be better appreciated after staining of the basal bodies, the organelles that anchor the cilia to the membrane, using γ-tubulin. In case of the whole mount, cells show a dispersed basal body pattern, whereas in the primary culture the basal bodies are highly concentrated at one point of the relatively large cell surface as delineated by co-staining for ß-Catenin (Figures 6E,F). ß-Catenin staining of cell-cell contacts reveals that the LIN41^+^ cells are arranged in a pinwheel structure both in whole mount and cultured cells. Importantly, the expression of the transcription factor FoxJ1, responsible for the ciliogenesis program (Yu et al., 2008), highly correlates with LIN41 expression both in the ventricle and primary culture (Figures 6G,H).

In addition to marker expression, an important test of the ependymal identity of the LIN41 positive cells in culture is the demonstration that they possess motile cilia. *In vitro* differentiated ependymal cells have been shown to initiate random beating after approximately 10 days in culture (Guirao et al., 2010). We therefore performed live cell imaging of the *in vitro* ependymal cultures from wild type tissue, and found that a beating tuft of apical cilia could be observed in the majority of the cells (Supplementary Video 1). This demonstrates that upregulation of *Lin41* coincides with the appearance of cells with functional motile cilia, an important confirmation that the ependymal differentiation program is faithfully modeled in this cell culture system.

## Discussion

### Lin41 is essential for mouse embryonic viability

The *Lin41* gene is highly conserved during animal evolution, from *C. elegans* to humans, and plays a major role in development. Its importance has been addressed in nematodes, fly, zebrafish and mouse, with loss of function experiments that result in developmental disruption and subsequent death of the animals (Slack et al., 2000; Lin et al., 2007; Löer et al., 2008; Maller Schulman et al., 2008). We developed a novel, independent gene trap mouse line, generated with insertion of a ß-Geo gene trap cassette after the second exon of the *Lin41* gene that eliminates wild type LIN41 expression in homozygous embryos (*Lin41^gt/gt^*) (Figure 1). Loss of function mutants in *C. elegans* cluster in the NHL domain (Slack et al., 2000), so that a C-terminal truncation of LIN41 is expected to result in a non-functional protein and a recessive mutational phenotype in mouse (Maller Schulman et al., 2008; Chen et al., 2012).

Using this line we obtained comparable results to those of Maller Schulman and colleagues, observing that embryos lacking *Lin41* did not survive past E9.5 or E10.5 (Figure 1) (Maller Schulman et al., 2008). This is in contrast to the line used by Niswander and colleagues, in which occasional embryos were retrieved at E14.5, indicating a somewhat more severe phenotype for our construct and genetic background (Chen et al., 2012). When isolated prior to E11.5, a neural tube closure defect was observed in all embryos, indicating 100% penetrance that is comparable to previous reports (Maller Schulman et al., 2008; Chen et al., 2012) (Figure 1C). These results reinforce the evidence of an essential role for *Lin41* during mouse development and for the correct execution of the neuroepithelial fusion events in neurulation. Observations in all published gene trap lines agreed on the lack of phenotype of the heterozygote animals, as they are in all cases viable, fertile and morphologically indistinguishable from their wild type littermates (Figure 1C). This suggests that, despite the reduced amount of protein due to a mutation in one *Lin41* allele, the remaining protein activity is sufficient to sustain normal development.

It should be noted that neural tube defects alone do not generally lead to embryonic lethality (Copp et al., 2003). The most common causes for mid-embryonic lethality are circulatory deficiencies, including failure of heart morphogenesis, alterations in extra-embryonic structures or placental insufficiency. *Lin41* is not expressed in the primordial heart of the embryos during the period of developmental arrest (see below), therefore, the cause of death is most likely related to placental insufficiency due to faulty development of the extra-embryonic structures (yolk sac, allantois or chorion) or defective trophoblast formation (Papaioannou and Behringer, 2012).

### Postnatal expression of *Lin41* in the lateral ventricle wall

Until now *Lin41* has been described as an early embryonic stem cell gene with only a few known areas of postnatal expression in adult stem cell niches such as the skin and testes (Rybak et al., 2009). But to date *Lin41* expression in the postnatal CNS has not been described. The mammalian brain has two major stem cells niches that persist in adulthood: the dentate gyrus of the hippocampus and the SVZ beneath the lateral walls of the lateral ventricles (Alvarez-Buylla and Lim, 2004). We used heterozygous (*Lin41^+/gt^*) gene trap animals to investigate postnatal *Lin41* promoter activity and found cells lining the ventricle walls to be positive (Figure 2A). Given the strong association between *Lin41* expression and stemness, we assessed the neurogenic capacity of these cells using a neurosphere culture assay. The neurospheres showed promoter activity for *Lin41*, but very low mRNA levels and no protein expression (Figures 2B–D). This disparity between the transcriptional activity seen with the *LacZ* reporter and the low level of endogenous *Lin41* mRNA and protein is most likely due to miRNA regulation of the endogenous transcript, leading to its translational inhibition and destabilization (Rybak et al., 2009). SVZ-derived neural stem cells, cultured as either neurospheres or in monolayer, express the let-7 miRNA and almost undetectable levels of *Lin41* mRNA (Rybak et al., 2009, F. Rehfeld, personal communication). This suggests that adult neural stem cells do not express functional LIN41 protein.

### Potential significance of LIN41 in the adult SVZ

If the stem cells are not responsible for LIN41 expression in the SVZ, the question remains, which cells are? One additional cell type in the stem cell niche is the ependymal cell. These multiciliated cells are important for the structure of the niche and function of the stem cells (Kuo et al., 2006; Lavado and Oliver, 2011; Paez-Gonzalez et al., 2011). We employed immunostaining on coronal brain sections and observed coexpression of LIN41 with the ependymal markers CD133 and CD24 in multiciliated cells (characterized by acetylated α-tubulin staining) (Figure 3). We confirmed these results using whole mount preparations with subsequent ß-Galactosidase reaction and LIN41 immunostaining to observe the pattern of *Lin41* promoter activity and protein expression throughout the lateral wall of the ventricle wall, respectively (Figure 4). To visualize the temporal expression pattern we used primary ependymal cell culture and found the onset of *Lin41* promoter activity to be around DIV8, consistent with the induction of all common ependymal markers at this time point (Figure 5). Comparison of immunostainings of the whole mount preparation with the ependymal culture, and the formation of motile cilia in the latter, confirms LIN41 co-expression with FOXJ1 in functional ependymal cells (Figure 6 and Supplementary Video 1). Although it is presently unknown if *Lin41* is under the transcriptional control of FOXJ1, these results suggest that LIN41 expression is upregulated in parallel with the ependymal differentiation program.

Given the known roles of LIN41 in pluripotent embryonic stem cells (Rybak et al., 2009; Chang et al., 2012), induced pluripotency (Worringer et al., 2013) and the inhibition of neural differentiation in the embryonic neural tube (Chen et al., 2012) it is perhaps surprising that LIN41 is expressed in ependymal cells and not in astrocytic type B cells which represent the bona fide stem cells in the SVZ (Temple and Alvarez-Buylla, 1999; Laywell et al., 2000). Although the stem cell potential of ependymal cells has been the subject of debate and differs between the spinal cord and brain, (Chojnacki et al., 2009; Sabelström et al., 2014) there is strong experimental support for the conclusion that the cortical ependymal lineage is terminally differentiated and post-mitotic. Nevertheless, adult ependymal cells express at least one additional pluripotency factor: *Sox2* (Ferri et al., 2004). Unlike *Lin41*, however, *Sox2* is also expressed by progenitors in the SVZ and dentate gyrus and is required for adult neurogenesis *in vitro* and *in vivo* (Ferri et al., 2004). Since *Sox2* is upregulated during the ependymal response to spinal cord injury (Lee et al., 2013), it might be informative to compare LIN41 expression and function in the brain and in the spinal cord in injury models. More generally, the presence of two pluripotency factors in ependymal cells suggests they may be inherently predisposed toward reprogramming. It would be interesting to study these cells in induced pluripotent stem (iPS) cell assays and determine if the correlation between *Lin41* expression and reprogramming efficiency seen in fibroblast model systems (Worringer et al., 2013) also applies to ependymal cells.

Another critical test will be to determine if *Lin41* is required for either ependymal specification or function. The final events controlling the fate of the radial glia at the end of embryonic neurogenesis, in particular as they relate to the onset of ependymal specification, have not been studied in detail. One exception is the homeobox transcription factor *Six3*, which is required for differentiation of ependyma and the suppression of radial glial features (Lavado and Oliver, 2011). Downstream targets for *Six3* have not yet been identified, but the core transcriptional program for ciliogenesis mediated by *RFX* family members working together with *FoxJ1* is required for ependymal morphogenesis (Choksi et al., 2014). *Lin41* expression might be involved in the initial burst of proliferation required to colonize the ventricular surface or in the transition to an epithelial cell physiology. The latter is consistent with the role of the Drosophila LIN41 homolog WECH in adherens contacts (Löer et al., 2008). WECH was shown to co-localize with the integrin adaptors TALIN and ILK to the cell membrane in muscle cells. This is in contrast to the cytoplasmic localization of LIN41 we see in ependymal cells and the known association of LIN41 to miRNA pathway proteins described in mES cells (Rybak et al., 2009; Chang et al., 2012).

At present we can only speculate on the functional consequences of *Lin41* expression in the adult SVZ. The ependymal cell culture system described in this study should be useful for the investigation of cell context specific functions for LIN41, and the reporter allele we have generated should facilitate lineage-tracing experiments studying the developmental origins of the ependyma and their response to injury. Our results identify the ependyma as an accessible cell system for targeted deletion of *Lin41* in adult animals.

## Author contributions

EC designed and performed the experiments, analyzed the data, designed the figures and wrote the manuscript. AR-W, AMR, and DTTN contributed to the experiment execution, critically reviewed and approved the final manuscript. FGW designed the experiments, analyzed the data and wrote the manuscript.

### Conflict of interest statement

The authors declare that the research was conducted in the absence of any commercial or financial relationships that could be construed as a potential conflict of interest.

## Supplementary material

Supplementary Figure 1 **(A,B)** Sagittal E10.5 and E13.5 *Lin41^+/gt^* mouse embryo sections, X-Gal stained. **(A)** X-Gal signal is confined to distal areas of the limb buds (arrowhead). **(B)** CNS appears X-Gal negative at E13.5. E. **(C)** Coronal section of P10 *Lin41^+/gt^* brain, showing lateral ventricle stained with X-Gal (scale bar 100 μm). **(D)** Coronal section of P12 *Lin41^+/gt^* brain, showing the hippocampus negative but the lateral ventricle positive for X-Gal (scale bar 100 μm). L, lateral; LV, lateral ventricle; M, medial.

Supplementary Video 1 **Recording of wild type ependymal primary culture after 15 days of differentiation, with cells displaying beating cilia**.
