# Supplementary figures and images for: Lin41/Trim71 is essential for mouse development and specifically expressed in postnatal ependymal cells of the brain
Source: Front Cell Dev Biol. 2015 Apr 2;3:20. doi: 10.3389/fcell.2015.00020 (PMC4382986; doi:10.3389/fcell.2015.00020)

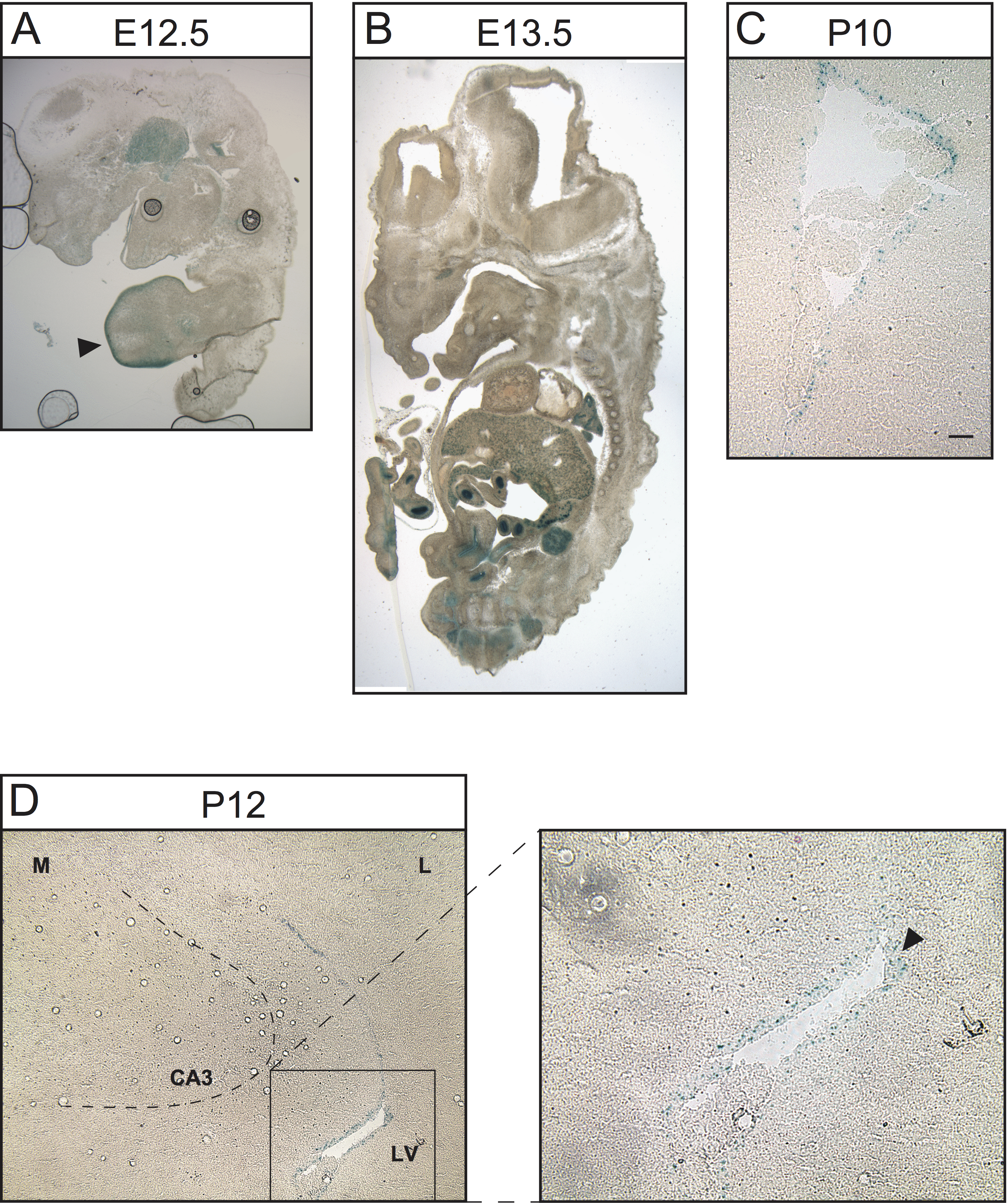

Supplement: Supplementary Figure 1 — (A,B) Sagittal E10.5 and E13.5 Lin41+/gt mouse embryo sections, X-Gal stained. (A) X-Gal signal is confined to distal areas of the limb buds (arrowhead). (B) CNS appears X-Gal negative at E13.5. E. (C) Coronal section of P10 Lin41+/gt brain, showing lateral ventricle stained with X-Gal (scale bar 100 μm). (D) Coronal section of P12 Lin41+/gt brain, showing the hippocampus negative but the lateral ventricle positive for X-Gal (scale bar 100 μm). L, lateral; LV, lateral ventricle; M, medial. [file Image1.JPEG]
